# Association of meal timing with dietary quality in a Serbian population sample

**DOI:** 10.1186/s40795-020-00375-2

**Published:** 2020-10-22

**Authors:** Zora Djuric, Marina Nikolic, Milica Zekovic, Melissa Plegue, Marija Glibetic

**Affiliations:** 1 Department of Family Medicine, Rogel Cancer Center, University of Michigan, Ann Arbor, MI 48109-1213 USA; 2 Center for Research Excellence in Nutrition and Metabolism, University of Belgrade, Belgrade, 11158 Serbia; 3 Present Address: European Food Safety Authority, Via Carlo Magno 1A, 43126 Parma, Italy

**Keywords:** Nutrition assessment, Serbia, Diet quality, Meal timing, EU recommendations

## Abstract

**Background:**

The world-wide adoption of Western lifestyles and eating patterns is associated with adverse effects on nutrient intakes. Here we evaluated the relationships between timing of meals and diet quality in Serbia, a Balkan country with a traditional eating pattern that includes the largest meal of the day as a late lunch.

**Methods:**

A dietary survey was done in the Republic of Serbia using a nationally-representative sample of 74 children and 260 non-pregnant adults. Nutrient intakes were calculated from two 24-h recalls. A Dietary Quality Score (DQS) enumerated how many European Union (EU) Science Hub recommendations were met for fruit and vegetables, fiber, saturated fat, sodium, and sugar. We evaluated whether the timing of dietary intakes is associated with DQS and body mass index.

**Results:**

The dietary intakes of children ages 10–17 and adults were similar and were high in total fat intake, with an average of 40% of energy from fat. Mean fruit and vegetable intakes of 473 g/day in adults exceeded the minimal EU recommendation. The most worrisome aspects of the Serbian diet were high intakes of saturated fat, sugar and sodium. Lunch was the meal with the highest mean content of energy, followed by breakfast and dinner, and the average time for lunch was 15:15. Consumption of a higher percentage of calories before 16:00 in adults was associated with higher fruit and vegetable intakes and with higher DQS. The subgroup of adults consuming their largest meal after 20:00 had a lower mean age, more men, and a larger percentage was employed outside of the home. There were no associations of meal timing with BMI, but the prevalence of obesity in this population sample was only 13%.

**Conclusions:**

These results indicate that an earlier meal pattern, and especially consuming the largest meal of the day earlier in the day, was associated with better quality diets. Public health efforts are needed to preserve nutrient intakes as the population shifts away from the traditional Serbian eating pattern. Long-term, deterioration of nutrient intakes could contribute to the increasing rates of obesity that have been observed in Serbia and world-wide.

## Background

One of the earliest reports describing diet in Serbia came from the Seven Countries study done more than 50 years ago. The Seven Countries study evaluated the relationships between diet and coronary heart disease in men. The Serbian diet was found to be between 8 and 10% saturated fat and yet the rate of cardiovascular diseases was low relative to that in other nearby locations with a similar saturated fat intake [1]. Currently, Serbia is classified as an upper middle income country with a developing economy, and economic development results in changing lifestyles [2]. Rates of obesity, an important risk factor for cardiovascular diseases, diabetes and cancer, are lower in Serbia than in the U.S. and many other upper-income countries, but obesity is increasing in Serbia [3–5]. Cardiovascular diseases and diabetes are now pressing public health problems in Serbia [6]. Cancer incidence is also increasing, as it is in most countries worldwide [7].

There is, however, little information available on dietary intakes in Serbia at the present time and what aspects of this diet impact on obesity. A limited number of published studies evaluated diets in children and adolescents in Serbia. These studies collected data six or more years ago and were mainly focused on children, on nutrition knowledge, and on frequency of consuming select foods without capturing the amount of intake [6, 8–10]. There is also limited dietary information from the Serbian National Health Survey of 2013. This survey collected information on disease prevalence, BMI, physical activity and eating habits in terms of foods eaten on a daily or weekly basis. Some improvements were noted since 2006, with decreasing intakes of animal fats, especially in urban areas, but whole grain bread intake decreased [6, 11]. This population-level data is valuable but a nation-wide, formal dietary assessment from which nutrient intakes can be calculated is lacking [12]. The recently established database for the nutrient content of Serbian foods will facilitate this assessment [13].

To establish the methodology for a large, nation-wide nutrition survey, we obtained pilot data on a nationally-representative sample of 334 individuals. The sampling methodology was designed to reflect the demographic distribution of the Serbian population as whole. Dietary data was collected using two un-announced 24-h recalls following the guidelines of the European Food Safety Authority and a standard demographic questionnaire [14, 15]. In this nationally-representative sample, we report the relationships between timing of meal intakes, BMI and major indicators of dietary quality: fruit, vegetables, fiber, saturated fat, sugar and sodium intakes.

The timing of the traditional Serbian eating pattern also afforded the opportunity to evaluate the impact of meal timing on diet quality and BMI. Traditionally, a late lunch is the largest meal of the day in Serbia, and dinner is typically a light meal. This meal pattern would result in consumption of a major portion calories earlier in the day than in Western cultures. It is, however, a meal pattern that is difficult to sustain when work patterns shift away from an early-day schedule. The objective of this study was to evaluate whether meal timing is related to obesity and diet quality in a Serbian population.

## Methods

### Subjects

The study was approved by the Ethics committee of the Institute for Medical Research, University of Belgrade (Dossier No. EO123/2017), and all procedures involving human subjects were in accordance with the Declaration of Helsinki. Written informed consent was obtained from each participant. In the case of children younger than 18 years of age, a parent gave consent and the child gave assent. The interviews were conducted between July and December of 2017.

The study sample was designed to be representative of the Serbian population as determined by the latest census from 2011 [16]. Sampling was stratified by age groups (10–17, 18–64, 65–74), by gender, and by region of residence. The regions of residence in Serbia were Vojvodina, South East Serbia, West Serbia and Belgrade. Recruitment was organized and conducted by project team members at the household level, with not more than one individual recruited per household. The survey plan was structured to capture a representative proportion of weekdays and weekend days for the study sample. Interviewers were advised to organize home visits to participants whenever possible. If it was not possible, another interview site was agreed upon depending on the region and location of the interviewer’s office. For the present analysis, pregnant women (*n* = 15) were excluded, resulting in a total of 334 non-pregnant subjects.

### Questionnaire

Two interviews were done with each subject, at least 1 week apart. The first interview consisted of collecting anthropometric data, administering a demographic questionnaire, and a 24-h dietary recall (see section on dietary assessment below). All of the initial interviews were done in person. Anthropometric data (height, weight) were measured for 65% of the study population. For the rest, self-reported values were collected. The study questionnaire collected demographic information on age, gender, marital status, place of residence, ethnicity, employment, education, smoking, height, weight, physical activity and health status.

Physical activity was assessed using the International Physical Activity Questionnaire short form that has been published [17]. This included questions on how much time was spent on moderate, vigorous and walking activities in the past 7 days (number of days and hours or minutes each time). For walking, only bouts of at least 10 min were enumerated. Time spent sitting was captured using questions that asked about time spent sitting on weekdays and on weekends. If a participant was not sure of time spent on physical activity, a time of 10 min/day was input, and this was needed for 23 of the questionnaires. The sitting and physical activity data was used to derive hours per week of activity or of sitting.

### Dietary assessment

The dietary assessment was conducted following the strict guidelines of the European Union (EU) Menu methodology as published previously [15]. In order to estimate usual dietary intakes, two non-consecutive 24-h recalls of diet were conducted with an interval of at least a week between the two recalls [14]. A multi-pass method was used to maximize accuracy of the recalls [15]. The multi-pass dietary recall utilized a visual for estimating serving sizes: a pictorial food atlas that was developed for the Balkan region to facilitate precise estimation of portion sizes as published by our group [14]. The first recall was always performed as a face-to-face interview and the second recall was conducted face-to-face or via phone, based on participant’s availability and preference.

Daily food and nutrient intakes were calculated using the Diet Assess & Plan software (DELTA Electronic Ltd., Subotica, Serbia). This is an advanced dietary assessment and nutrition planning software tool that has been used previously in national, regional and international nutritional surveys, and it was evaluated in the European Food Safety Authority (EFSA) ring trial involving six countries [18–20]. Nutrient intake calculations were performed using the Serbian Food Composition Database [13]. As published previously, the database was harmonized with European Food Information Resource Network of Excellence (EuroFIR) standards and embedded in the EuroFIR platform and represents the core element of the Balkan Food Platform [13]. Average daily intakes for each subject were derived as a mean of the two 24-h recalls.

### Meal timing

The time of each eating occasion reported during the 24-h dietary recall was recorded. The time noted represents the start of the eating occasion. This varied across the 2 days of intake, and the times for each eating occasion for each subject across the 2 days of intake were averaged. The eating occasions were denoted in the data output as before breakfast, breakfast, morning snack, lunch, afternoon snack, dinner, and after dinner snack. For some subjects, there were two afternoon snacks, resulting in a maximum of eight eating occasions that were recorded for each subject. This data was used to calculate average calories consumed by each subject before 16:00.

### Age categories

Five age categories were created to evaluate differences in diet and BMI by age. The first category was for children ages 10–17. Adults ages 18–64 were categorized into tertiles. Since some people were of the same age, the tertiles are not precisely equal in number. The last category was for adults ages 65–74 years since the retirement age in Serbia is 65 years.

### Diet Quality Score (DQS)

We computed a DQS for adults to evaluate how many dietary recommendations were being met by each adult subject. This analysis focused on adults only since the number of children in the sample was smaller, dietary recommendations in children differ from that in adults, and health issues are different in children than in adults. The DQS summed the number of dietary recommendations met by each subject.

The dietary recommendations vary from country to country and change over time. We focused on those dietary factors for which healthy eating recommendations have been established as summarized by the European Union (EU) Science Hub of the European Commission [21]. The DQS therefore included one point for meeting each of the EU Science Hub recommendations for fruits and vegetables, fiber, saturated fat, sugar, and sodium as detailed below, resulting in a score of 0–5.

For fruit and vegetable intakes, recommendations have ranged from 5 servings a day to more than 13 per day, given in ½ cup servings [21, 22]. The World Health Organization in 2006 recommended a daily intake of fruit and vegetables of at least 400 g/day [23]. Using an estimate of 85 g vegetables or fruit in a ½ cup serving, 400 g/day is about 5 servings/day. Intakes of 400 g/day or higher therefore received one point. Fiber recommendations are typically 25–35 g/day, often provided in a sex-specific and age-specific manner. An adequate intake for both children and adults is 14 g/1000 kcal in the Dietary Guidelines for Americans, which harmonizes well with the European recommendations [21]. We therefore awarded one point for meeting or exceeding an intake of 14 g fiber/1000 kcal.

Recommendation for sugar intake generally include consuming less than 10% of energy from added sugars, and we used that recommendation for awarding a point in the DQS [21]. For fat intake, moderate intakes of total fat (20–35% of energy) have been recommended in the past, but more recent evidence shows little associations of total fat intake with disease risks [21]. The recommendations have been more consistent for limiting saturated fat, and we therefore used less than 10% of calories from saturated fat as the cut-off to award a point in the DQS. For sodium intake, recommendations are to limit intake to 2.0–2.4 g/day, and we awarded a point towards the DQS for sodium intakes less than 2.3 g/day [21].

Whole grain intake was not available from our data set and was not used as a criterion, but this likely does overlap with fiber intake which we did include. Protein and water intakes also were not used in the score. An average requirement of 0.60–0.66 g protein/kg body weight has been set as a recommendation across countries [21]. In our population sample, only 14 subjects (5 children and 9 adults) reported consuming less than 0.6 g/kg protein, and the mean was 1.1 g protein/kg for adults. Protein intake therefore was not used as criterion in the DQS. Recommendations for water intakes have not been widely made and the recommendations vary from 1000 to 2700 ml/day [21]. This made it difficult to select a scoring criterion for water intake and it was omitted from the DQS.

To generate a continuous DQS, we constructed a Diet Quality z-Score. Normalized intakes for each nutrient or food were calculated as the difference between each subjects’ value and the recommended value divided by the standard deviation. The differences were taken using positive values for dietary intakes that exceed the recommendations for fiber (14 g/1000 kcal) and fruit/vegetable intakes (400 g/day), and negative values for dietary intakes that exceeded the recommended limit for saturated fat (10% of energy), sodium (2300 mg/day) and sugar (10% of energy). The Diet Quality z-Score was calculated by summing the five normalized nutrient or food scores.

### Statistical analyses

Data from the demographic questionnaire and from the nutrition analysis program was maintained in Excel. All statistical analyses were conducted in IBM SPSS Statistics for Windows, Version 24.0. (IBM Corp., Armonk, NY). Comparisons between groups (adults versus children, or early versus late eaters), were made using two-sample, two-sided t-tests for continuous variables or by using Pearson Chi-square tests for categorical variables. Correction of *p*-values for false discovery rates (FDR) was done using the method of Benjamini and Hochberg [24]. Trends of DQS versus percent of calories consumed before 16:00 were explored using linear regression models, adjusting for participant age and gender. Comparisons of energy intakes in three ordinal categories of DQS, defined by DQS scores of 0–1, 2–3, or 4–5, were evaluated using ANOVA, and natural log transformation of the data to achieve normality was used for the kcal/day variable. The Tukey honest significance test was used post-hoc to control for multiple comparisons.

To evaluate the effects of age category and gender on dietary intakes, linear regression models for each of the key dietary intake outcomes were constructed (fruit and vegetables, fiber, saturated fat, sodium and sugar). These models included age category, gender and the interaction between age and gender as predictors. When the interaction was not significant it was removed to assess the main effects of age category and gender.

**Fig. 1:**
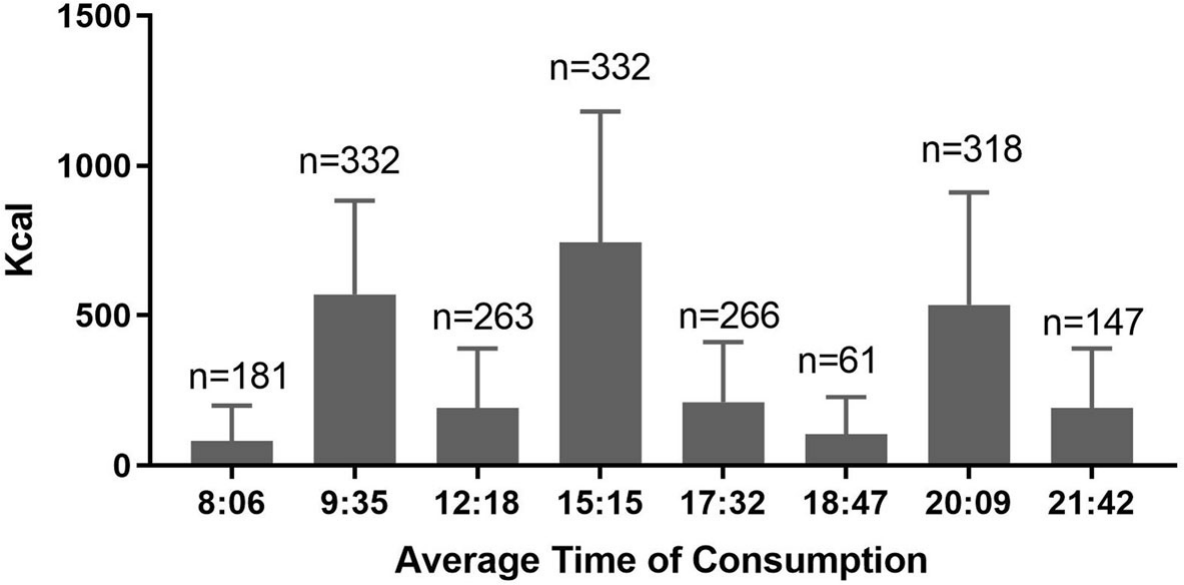
Mean energy intakes (with SD) across eating occasions in 334 adults and children. Breakfast was at about 9 am, lunch at 3 pm and dinner at 8 pm. The other eating occasions were reported as snacks. For both children and adults, linear mixed models indicated that lunch had a significantly higher calorie content than either breakfast or dinner, and there were no significant differences in calorie content between breakfast and dinner

Calorie content across the three main meals, breakfast, lunch and dinner (Fig. 1), was compared using linear mixed models with kilocalories per meal as the outcome. The model included meal as a categorical covariate and a random intercept was used to account for within person correlation. Separate models for children and adults were constructed, and an overall model with an interaction between age group and meal was also investigated. We also calculated the timing of the largest meal of the day, averaged across 2 days of assessment for each subject, based upon energy content of the reported meals. Histograms were constructed to evaluate the timing of when the largest meal was consumed by adults who and do not work outside the home. Reasons for not working outside the home included being retired, currently unemployed, disabled or working as a homemaker.

## Results

### Subject characteristics

There were 349 study participants who had complete data for both diet and demographics. For the present analysis of diet quality, we removed 15 pregnant subjects. This resulted in a data set of 74 children and 260 adults. The vast majority of respondents self-identified as being Serbian (90%); 10 subjects were Montenegrian, 3 Croatian, 1 Hungarian, and 8 specified their ethnicity as “other”. The surveys were carried out in the calendar year 2017, and most were completed in the fall (85%), with an additional 11% in the summer.

Of the 334 subjects, 74 reported having a chronic health condition at the present time: 35 with cardiovascular diseases or hypertension, 9 respiratory conditions, 5 diabetes, 3 pre-diabetes, 4 hypothyroidism, 3 mental health issues, and 15 other health issues. Ten of these individuals reported consuming a diet that was altered in some way due to their health condition (mean age 51 years, 5 females). Eight other individuals reported following a weight loss diet and 16 individuals reported following a vegetarian diet. Twenty-seven adult subjects (10% of all adults) reported consuming more than 2 drinks/day, on average.

As shown in Table 1, 90% of the study participants lived in areas classified as urban cities. Urban/rural residence did not differ by participant age category (*p* = 0.55). Education was classified according to International Standard Classification of Education 97 system. The proportion of study participants with some type of education beyond a high school diploma (either at least some college or a technical school) did not differ significantly by adult age category but it was lowest for adults in the oldest age category. The prevalence of overweight or obesity was higher in the oldest adult age category, as shown in Table 1.

**Table 1:** Characteristics of subjects in each age category. Data shown are the mean and SD or the percentage of subjects within each age category with the indicated characteristic

Age category	Number ^a^	Age, years (mean and SD)	Serbian ethnicity ^b^ (n, %)	Female (n, %)	Current Smoker (n, %)	Overweight or Obese ^b^ (n, %)	Post-secondary education ^c^ (n, %)	Employed (n, %)	Student (n, %)
10–17	74	14 (2)	66 (92%)	37 (50%)	4 (6%)	5 (7%)	0	0	67 (92%)
18–29	75	25 (3)	72 (97%)	40 (53%)	26 (35%)	20 (27%)	43 (59%)	38 (51%)	26 (35%)
30–47	73	38 (6)	64 (89%)	40 (55%)	24 (35%)	29 (41%)	50 (70%)	62 (87%)	2 (3%)
48–64	73	55 (4)	67 (93%)	43 (59%)	25 (35%)	43 (59%)	44 (61%)	50 (69%)	0
65–74	39	69 (3)	32 (82%)	19 (49%)	7 (19%)	26 (70%)	18 (46%)	3 (8%)	0
All	334	37 (19)	301 (92%)	179 (54%)	86 (27%)	123 (38%)	155 (48%)	153 (46%)	95 (29%)

^a^ A small portion of subjects had missing data for one or more factors: *n* = 14 for smoking status, *n* = 7 for body mass index, *n* = 12 for education, *n* = 4 for employment status and *n* = 5 for ethnicity. The percentages shown in each column are for subjects who had valid data for that category

^b^ The proportion of subjects differed across the four adult age categories for the indicated variables, as determined by Pearson Chi square tests. These tests evaluated differences in ethnicity, smoking, and overweight/obesity by adult age category, and both ethnicity and overweight/obesity prevalence differed by adult age category (*p* = 0.04 for ethnicity and *p* < 0.001 for overweight/obesity)

^c^ Post-secondary education includes individuals who completed at least some college or technical training beyond high school (International Standard Classification of Education 4). In the total population above age 18, only 8% did not finish high school (International Standard Classification of Education 0, 1 or 2)

### Overweight and obesity

Body weight and height was measured by study interviewers for 65% of the study sample, and the remainder of this data was from self-report. Body weight and height was missing for 6 subjects. Body mass index (BMI) was calculated as kg/m^2^. Of 254 adults with complete weight and height data, 34 were obese with BMI > 30 kg/m^2^ (13%) and 84 were overweight (BMI > 25 kg/m^2^ and < 30 kg/m^2^, 33%). In adults, Spearman correlations of BMI with DQS, percent of calories consumed before 4 pm, the number of eating occasions/day, minutes/day spent sitting, and with minutes/week spent on moderate and vigorous activity were not statistically significant. Adult BMI was positively correlated with age, Spearman ρ = 0.401, *p* <  0.001. These results were similar when restricted to data using only the 151 subjects with measured BMI values: 11% were obese, 33% were overweight, and BMI was correlated with age (Spearman ρ = 0.358, *p* <  0.001) but not with physical activity or time spent sitting.

### Dietary intakes

Table 2 shows mean dietary intakes in the Serbian population sample to facilitate comparison to dietary recommendations compiled by the European Commission [21]. Mean fruit and vegetable intakes were above the minimal recommendation of 400 g/day, and the range of intakes reported was 15–1783 g/day (Table 2). All study participants had at least some intake of fruits and vegetables, all the children and all except two adults had some vegetable intake, and 23% of children and 20% of adults had no fruit intake. Mean water intake of 2191 ml/day also was within the recommendations, although the recommendation made span a wide range of 1000–2700 ml/day for persons over the age of 14 [21]. Total fluid intakes from water and all other beverages other than liquor were higher: 2653 ml/day for children and 3027 ml/day for adults. Fiber was derived mainly from three groups of foods: fruits and vegetables (38%), grains (33%), and seeds and nuts (12%). Mean fiber intake reached the recommendation for consuming at least 14 g/1000 kcal only in adults of ages 65–74..

**Table 2:** Nutrient intakes in the Serbian population sample. Data shown is mean (SD)

Nutrient	Children (ages 10–17) *n* = 74	Adults (ages 18–74) *n* = 260	All subjects, *n* = 334
Energy (kcal/day)	2280 (951)	2265 (987)	2268 (978)
Carbohydrate (% of energy)	46 (8)	44 (10)	44 (10)
Protein (% of energy)	15 (4)	16 (4)	15 (4)
Fat (% of energy)	39 (8)	40 (9)	40 (9)
Saturated Fat (% of energy)	12 (4)	13 (4)	13 (4)
Fiber (g/1000 kcal)	8.1 (3.4)	10.9 (6.4) ^a^	10.3 (6.0)
Fruit (g/day)	169 (173)	184 (188)	181 (184)
Vegetables (g/day)	256 (200)	289 (207)	281 (205)
Fruit and Vegetables (g/day)	424 (259)	473 (295)	463 (288)
Sodium (g/day)	3.66 (2.69)	3.99 (3.00)	3.92 (2.93)
Sugar (% of energy)	13.6 (6.4)	13.2 (7.4)	13.3 (7.2)
Water (ml/day)	1996 (1327)	2246 (1333)	2191 (1334)
Protein, g/kg body weight	1.45 (0.56)	1.11 (0.46) ^a^	1.19 (0.51)
Energy density (kcal/g food)	1.01 (0.34)	0.89 (0.32) ^a^	0.92 (0.32)
% of energy consumed before 16:00	59 (16)	61 (18)	60 (18)
Number of eating occasions per day	6.1 (1.0)	6.1 (1.1)	6.1 (1.1)

^a^ Marked variables differed significantly between children and adults by two-sample t-tests, with *p* < 0.001 for both fiber and protein, and *p* = 0.006 for energy density, each of which remained significant after adjustment for false discovery rates

The mean intake of total fat at 40% of energy was higher than what is considered to be a moderate range, generally defined as 20–35% of energy from total fat [21]. Saturated fat intake was a mean of 13% of energy in this study population (below 10% of energy is recommended), and sugar intake was a mean of 13% of energy (below 10% of energy is recommended). Sodium intake was very high with a mean of 3.9 g/day (less than 2.3 g/day is generally recommended).

### Diet Quality Score (DQS)

The DQS was a simple sum of the number of EU dietary recommendations that were met for five items: fruits and vegetables, fiber, saturated fat, sugar, and sodium. One point was awarded for meeting the recommendation in each of the five categories. Whole grain intake in servings was not available in this diet analysis, but 33% of dietary fiber in this study sample was obtained from total grain intake. Only 12 adults and one child had a score of 5, and 78% of adult subjects had scores of 3 or lower.

Among the individual components of the DQS, fruit and vegetables intakes and fiber were met by about half the subjects, but the numbers meeting the sugar, sodium and saturated fat recommendations were much lower. Among the 260 adult subjects, there were 139 with at least 400 g/day fruits and vegetables, 130 with at least 14 g fiber/1000 kcal, 90 with less than 10% of energy from sugar, 66 with 2.3 g or less of sodium/day, and 58 with a diet < 10% of energy from saturated fat.

A Diet Quality z-Score was also calculated as a continuous variable using the difference in intake versus the recommendation divided by the standard deviation for each of the five factors as described in Methods. This z-like score takes into account that some intakes might be closer to the recommended levels than others without necessarily meeting the recommendation. Women had significantly higher average DQS z-scores than men (2.7 vs. 2.0, *p* < 0.001, z-score − 1.5 v.-2.5, *p* < 0.001), and z-scores increased with age (Spearman ρ = 0.284, *p* < 0.001).

### Timing of meals

The average time for all eating occasions recorded in children and adults is shown in Fig. 1, along with the number of subjects who reported consuming each meal or snack. The average calorie content of meals and snacks is shown for those study participants who reported consuming food on each occasion. All children and almost all of the adults reported eating breakfast, lunch and dinner. There was an average of six eating occasions per day for both adults and children (Table 2). The average calorie content of snacks was smaller than that of the daily meals, and a smaller number of study participants reported eating snacks than meals, as shown in Fig. 1.

For both children and adults, lunch had a significantly higher mean calorie content than both breakfast and dinner. In children, the marginal mean of 665 kcal (SE 38) for lunch differed from both the 560 kcal (SE 38) for breakfast with *p* = 0.025 and the 520 kcal (SE 38) for dinner with *p* = 0.002. In adults, the marginal mean of 761 (SE 24) kcal for lunch differed from the 537 kcal (SE 24) for breakfast with *p* < 0.001, and from the 568 (SE 24) kcal for dinner with *p* < 0.001. The average calorie content between breakfast and dinner was not significantly different in either group (*p* = 0.41 for children and *p* = 0.29 for adults). For comparing the calorie content of the three meals using data for children and adults combined, linear mixed models showed that adult status did not have a significant interaction with meal calorie content (*p* = 0.28). The average time lunch was consumed across all subjects was 15:15. Lunch was the largest meal of the day for 47% of adults. In evaluating individual data for adults, 84% consumed lunch within 1 h of the day on the two different days that recalls were done, and on average the difference in timing between the 2 days was 24 min (SD 54 min).

We next evaluated whether the timing of energy intakes was associated with BMI, age, gender and measures of dietary quality (Table 3). For adults, the mean percentage of calories consumed by 16:00 was 60%. Using two-sample t-tests, adults who consumed at least 60% of their daily energy intake by 16:00 (4 pm), versus those that did not, were older (mean age 47 vs. 40 years), and consumed greater amounts of fruits and vegetables (mean 521 v. 425 g/day). Early eaters also consumed less total calories per day (2135 v. 2402 kcal/day), but this was not significant after adjusting for multiple comparisons, and mean DQS was higher although not significantly so (Table 3). Fruit and vegetable intakes were the main statistically significant difference in diet quality between early and late eaters. Other components of the DQS did not differ significantly between the two groups (Table 3).

**Table 3:** Characteristics of adult subjects who did or did not consume at least 60% of their daily energy intake by 16:00

Characteristic	Early eaters *N* = 133	Late eaters *N* = 127	*P*-value
Percent of calories consumed by 16:00	75 (9)	46 (12)	< 0.001 ^a^
Total calorie intake, kcal/d	2135 (880)	2402 (1076)	0.029
Age, years	47.1 (16.6)	39.6 (15.2)	< 0.001 ^a^
Female, number and %	72 (51%)	70 (48%)	0.874
Employed outside the home, number and %	78 (59%)	75 (59%)	0.764
BMI, kg/m^2^ (all data)	25.7 (3.7)	24.8 (4.4)	0.111
BMI, kg/m^2^ (measured only, *n* = 83 early, *n* = 68 late eaters)	25.6 (3.6)	24.5 (4.2)	0.103
Overweight or obese, number and %	51 (38%)	67 (53%)	0.153
Number of eating occasions/day	6.1 (1.1)	6.2 (1.0)	0.733
Energy density (kcal/gram food)	0.87 (0.28)	0.92 (0.35)	0.219
Fruits and vegetables, g/day	521 (332)	425 (242)	0.008 ^a^
Fiber, g/1000 kcal	11.7 (7.1)	10.2 (5.5)	0.065
Saturated fat, % of energy	13.3 (4.7)	13.3 (4.3)	0.893
Sugar, % of energy	13.4 (8.3)	13.1 (6.2)	0.748
Sodium, g/day	4.1 (3.6)	3.9 (2.3)	0.636
Diet Quality Score of 4–5, number and % ^b^	40 (30%)	26 (21%)	0.075
Diet Quality Z-score ^c^	−1.76 (2.48)	−2.23 (1.87)	0.089

^a^ The *p*-values shown are from two-sample t-tests or from the Chi-square test for gender and number of people with a Diet Quality Score of 4 or 5. After adjustment for false discovery rates, *p* < 0.01 remained statistically significant

^b^ The Diet Quality Score (DQS) enumerates how many EU dietary recommendations were met on a scale of 0 to 5. The five dietary recommendations included in the score were fruit and vegetable servings, saturated fat, sodium, sugar and fiber

^c^ Each component of the DQS was z-transformed and summed to create a continuous variable

The standardized Diet Quality z-Score was weakly associated with the percentage of calories consumed by 16:00, with β = 0.03 (SE 0.007), *p* < 0.001. Every 10% increase in calories consumed by 16:00, the Diet Quality z-Score increased by a modest 0.3 in an unadjusted regression model. When age and gender were also included in the model, the results were similar β = 0.02 (SE 0.007), *p* = 0.004. Age and gender were also related to the Diet Quality z-Score with increased age associated with higher scores and men having lower scores. Mean Diet Quality z-Scores were − 2.52 and − 1.55 for men and women, respectively.

#### Timing of the largest meal of the day

**Fig. 2:**
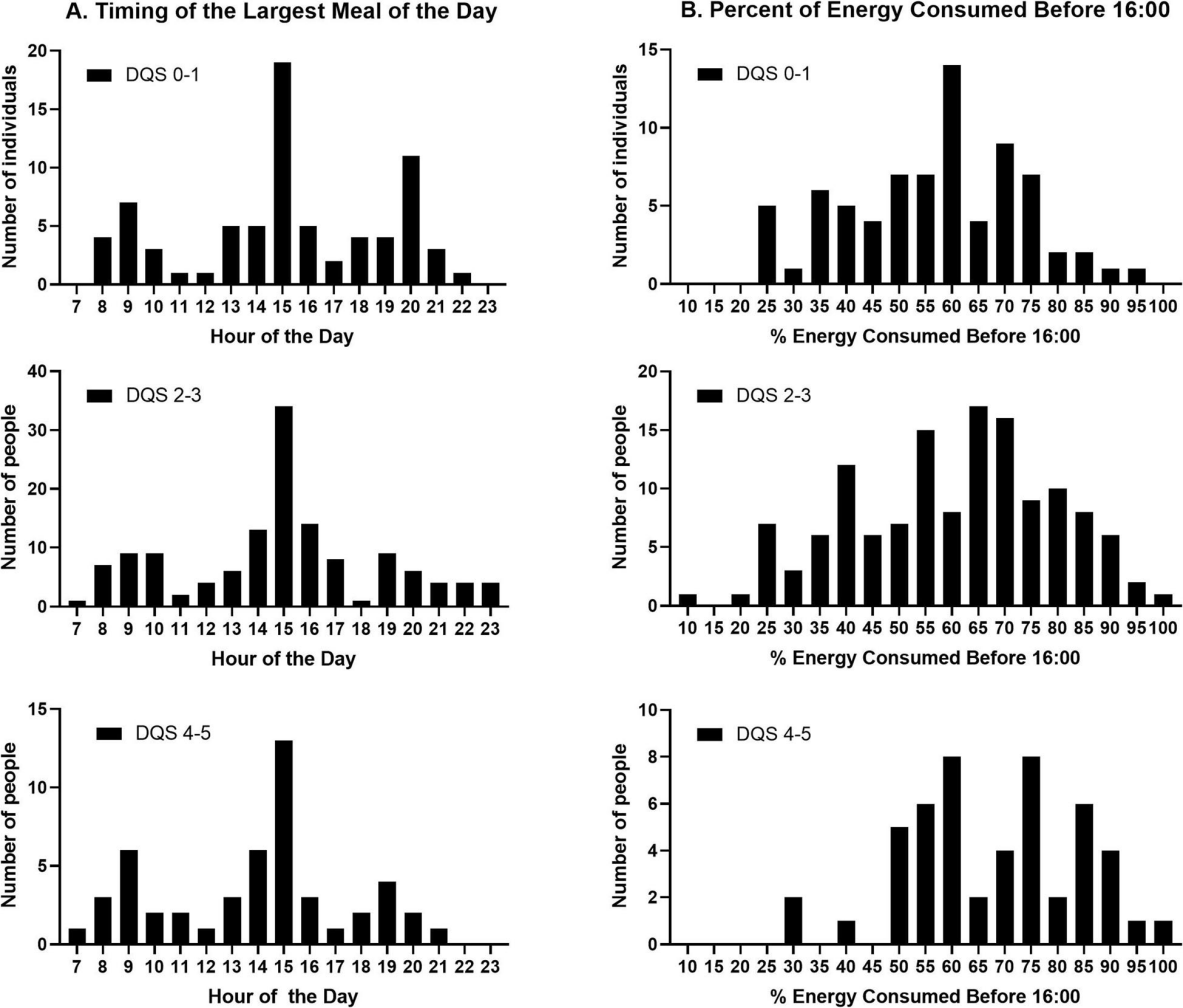
Timing of dietary intakes in adults who met 0–1, 2–3, or 4–5 of the EU dietary recommendations, indicated as Dietary Quality Scores (DQS). Data shown is number of adults as a frequency distribution for: A. Time at which the largest meal of the day was consumed and B. Percent of energy consumed before 16:00. Mean energy intake was lower, and percent of energy consumed before 16:00 was higher, in adults with DQS scores of 4 or 5 versus the other two categories of DQS scores, as given in the Results

Since there was a fair amount of variability in energy intakes across meals and breakfast was a sizable meal (Fig. 1), we sought to identify the timing of the largest meal of the day in more detail. The largest meal was defined as the meal with the highest caloric content. The histograms showing time of consumption of the largest meal among 260 adults stratified by DQS are in Fig. 2. The group with DQS of 4–5 displayed a lower frequency of consuming the largest meal after 20:00 and a higher frequency of consuming at least 60% of energy before 16:00. Of 50 individuals with DQS of 4–5, 72% consumed 60% or more of their daily energy before 16:00, and this occurred less frequently for adults with DQS of 0–1 (35 of 75, 47%) or DQS of 2–3 (58 of 135, 43%), as shown in (Fig. 2).

The mean percent of energy consumed before 16:00 in adults with DQS of 0–1 (category 1), DQS of 2–3 (category 2), and DQS of 4–5 (category 3), were 56.6% (SD 16.5), 60.1% (SD 18.8) and 68.1% (SD 16.1), respectively. ANOVA analyses with the Tukey post-hoc tests indicated that the percent of energy consumed before 16:00 was higher in DQS category 3 than in DQS category 2 or 1 with *p* < 0.02 in either case. Mean energy intakes were 2528 (SD 1031, *n* = 75), 2337 (SD 998, *n* = 135) and 1677 (SD 587, *n* = 50) kcal/day in the DQS categories of 1, 2 and 3, respectively. After natural log transformation to normalize the data, ANOVA analyses with the Tukey post-hoc tests indicated that mean energy intake per day in DQS category 3 was significantly lower than in the two other categories with *p* < 0.001 in either case.

Working outside the home did yield a later distribution of when the largest meal of the day was consumed, as shown in Table 4. However, the average hour for consuming the largest meal was similar (15:15 versus 14:56 for working and non-working adults, respectively, *p* = 0.524). The frequency of consuming at least 60% of energy before 16:00 also was similar in working and non-working adults (Table 3).

**Table 4:** Characteristic of Adults Who Do or Do Not Report Eating After 20:00. Data shown is mean and SD or number and percent for 260 non-pregnant, adults

Characteristic	Consume Food After 20:00, *n* = 193	Do Not Eat After 20:00, *n* = 67	*P*-value^a^
Age, years	41.6 (15.6)	48.8 (17.3)	0.002^a^
Female, number and %	98 (51%)	44 (66%)	0.035^a^
Smoker, number and %	117 (63%)	52 (81%)	0.006^a^
Employed outside the home, number and %	121 (63%)	32 (49%)	0.034^a^
BMI, kg/m^2^ (measured only, *n* = 114 and *n* = 37, respectively)	25.1 (4.0)	25.0 (3.8)	0.832
BMI, kg/m^2^	25. 1 (4.0)	25.7 (4.3)	0.334
Overweight or obese, number and %	84 (44%)	34 (51%)	0.273
Percent of Energy consumed before 16:00	57 (18)	70 (15)	< 0.001^a^
Total calorie intake, kcal/d	2407 (1011)	1857 (787)	< 0.001^a^
Energy density (kcal/g food)	0.91 (0.33)	0.84 (0.28)	0.108
Number of eating occasions/day	6.1 (1.1)	6.2 (1.1)	0.896
Fruits and vegetables, g/day	464 (289)	501 (311)	0.374
Fiber, g/1000 kcal	10.4 (6.8)	12.4 (5.1)	0.030^a^
Saturated fat, % of energy	13.8 (4.5)	11.9 (4.1)	0.002^a^
Sugar, % of energy	12.3 (6.1)	15.9 (9.7)	0.001^a^
Sodium, g/day	4.2 (3.1)	3.2 (2.7)	0.020^a^
Diet Quality Score 4–5 ^b^, number and %	36 (19%)	30 (45%)	< 0.001^a^
z-Diet Quality Score ^c^	−2.2 (2.1)	−1.4 (2.5)	0.009^a^

^a^ *P*-values are from the Person Chi-square for categorical variables or from two-sample t-tests for continuous variables. After correction for False Discovery Rates, *p* < 0.036 was significant, as marked

^b^ The number of individuals with a Diet Quality Score (DQS) 4 or 5 is shown. The range of DQS was 0-5

^c^The z-Diet Quality Score was calculated as the sum of z-scores for each of the five diet quality criteria

## Discussion

In this population-based sample of individuals residing in Serbia, we assessed the impact of meal timing on obesity and diet quality. On average, the EU recommendation for fruits and vegetables was exceeded, and the average intake in g/day was above the mean of almost 300 g/day that was reported for fruits and vegetables globally in a review of diet survey from 113 countries during 2010 [25]. At the individual level, however, about half of the study population met the fruit and vegetable recommendations. This was also true for fiber intake (Results). Mean intakes of total and saturated fat, sugar and sodium, however, were all above the desired ranges with only 22–34% of the sample meeting the recommendation for each of these nutrients [21]. Despite this, the prevalence of overweight and obesity combined was only 45% for this sample of Serbian adults. Serbia as whole does have lower rates of overweight and obesity than Western countries such as the U.S. where 72% of the population is overweight or obese [5]. The prevalence of obesity in this Serbian population sample surveyed in 2017 was only 13% which is dramatically less than that in the U.S. where 40% of the population was obese in 2015–2016 [5, 26]. Unfortunately, obesity rates are increasing in Serbia as they are world-wide [3–5].

One interesting characteristic of the traditional Serbian diet is that lunch is the largest meal of the day. Lunch was consumed, on average, in the middle of the afternoon at 15:15 (Fig. 1). In Serbia, the traditional lunch is a large, cooked meal with soup followed by meat and vegetables. Other meals are rarely cooked and instead typically consist of bread, cheese, processed meats, salad, or ready-made foods purchased from a bakery such as the traditional filo and cheese dish, gibanica [27]. How this eating pattern may be changing as the economy develops and Western living patterns are adopted is not yet defined. Meal timing is also a part of the so-called “nutrition transition” away from culturally-traditional eating patterns [28]. Globally, the timing of meals across countries does vary, and it also appears that early eating could be helpful for weight control [29, 30]. In this sample of Serbian adults, meal timing was associated with diet quality but not with obesity. An association with obesity may emerge, however, if the trends away from the traditional eating pattern with declining diet quality become more prevalent and persist over time.

Many studies have shown that eating later in the day is associated with obesity and adult weight gain [31–33], although not all studies agree [34]. Consuming more calories earlier in the day also could allow for a longer fast overnight, and extending the overnight fast is reported to improve metabolic health [35, 36]. In addition, consuming breakfast daily has emerged as a strategy to avoid weight gain [36–38]. Results of the present study are consistent with these findings since eating a larger percentage of calories by 16:00 and completing food consumption before 20:00 was associated with better diet quality.

In agreement with our data, the Serbian National Health Survey of 2013 showed that the proportion of females consuming fruits and vegetables on a daily basis was higher versus that in males [11]. The reported intakes of fruits and vegetables overall were lower than the average for Europe, and more than half of the Serbian population was found to either not eat fruit or eat fruit rarely based on a diet questionnaire [11]. In our study that was completed in 2017, only one-fifth of the sample had no fruit intake and every individual consumed at least some fruits and vegetables across the 2 days of assessment. Mean intake of fruits and vegetables was above the EU recommendation. With the recall method we used and formal analysis of all foods eaten, small amounts of fruits and vegetables in mixed dishes are enumerated and this might not be readily quantified using a questionnaire.

Limitations of the present study are that diet and health behaviors were self-reported. There is evidence that persons who have overweight or obesity under-report dietary intakes [39, 40]. We maximized accuracy by using a multi-pass method for the dietary recalls and by following strict EFSA guidelines for nutrition assessment, but this limitation should be recognized. Most of the surveys were carried out in the fall, and dietary intakes do vary somewhat by season, with lowest calorie intakes reported in the winter [41]. In addition, some of the BMI data was by self-report, but mean BMI and the associations that we evaluated with BMI were similar with and without the self-reported data. In a review of studies that addressed the accuracy of self-reported weight and height, self-reported BMI was found to range 0–2.2 BMI units lower than measured BMI [42]. Strengths of the study include that the population sample was designed to reflect the Serbian population as a whole, the data add to the very limited information available about Serbian diets, and that the timing of all eating occasions was captured such that the effect of meal timing on diet quality could be evaluated.

## Conclusions

The major findings are that the dietary factors of greatest concern in the Serbian diet were saturated fat, sugar and sodium. Half the sample met with EU recommendations for fruits and vegetables and for fiber. An earlier meal pattern, and especially consumption of the largest meal of the day earlier in the day, was associated with better quality diets. A later meal pattern conversely represents a departure from the traditional Serbian eating pattern and was more prevalent in younger individuals, men and working adults. Shifting meal timing to accommodate changes in lifestyles may be a necessity, but efforts are needed to find ways to improve dietary quality while changes in meal consumption take place.

## Data Availability

The datasets analyzed during the current study are available from the senior author on request for data presented in aggregate to protect anonymity of subjects.

## Abbreviations

BMI: Body mass index
DQS: Diet Quality Score
EFSA: European Food Safety Authority
EU: European Union
EuroFIR: European Food Information Resource Network of Excellence
SE: Standard error
